# Response of Fungal Sub-Communities in a Maize-Wheat Rotation Field Subjected to Long-Term Conservation Tillage Management

**DOI:** 10.3389/fmicb.2022.829152

**Published:** 2022-03-29

**Authors:** Cunzhi Zhang, Hao Liu, Senlin Liu, Sarfraz Hussain, Liting Zhang, Xiaowei Yu, Kaixun Cao, Xiuli Xin, Hui Cao, Anning Zhu

**Affiliations:** ^1^Key Laboratory of Agricultural Environmental Microbiology, Ministry of Agriculture and Rural Affairs, College of Life Sciences, Nanjing Agricultural University, Nanjing, China; ^2^Key Laboratory of Integrated Regulation and Resource Development on Shallow Lakes of Ministry of Education, College of Environment, Hohai University, Nanjing, China; ^3^Fengqiu Agro-Ecological Experimental Station, State Key Laboratory of Soil and Sustainable Agriculture, Institute of Soil Science, Chinese Academy of Sciences, Nanjing, China

**Keywords:** conservation tillage, fungal sub-communities, assembly process, community structure, co-occurrence pattern

## Abstract

Conservation tillage is an advanced agricultural technology that seeks to minimize soil disturbance by reducing, or even eliminating tillage. Straw or stubble mulching in conservation tillage systems help to increase crop yield, maintain biodiversity and increase levels of exogenous nutrients, all of which may influence the structure of fungal communities in the soil. Currently, however, the assembly processes and co-occurrence patterns of fungal sub-communities remain unknown. In this paper, we investigated the effects of no-tillage and straw mulching on the composition, assembly process, and co-occurrence patterns of soil fungal sub-communities in a long-term experimental plot (15 years). The results revealed that combine straw mulching with no-tillage significantly increased the richness of fungi but not their diversity. Differential abundance analysis and principal component analysis (PCA) indicated that tillage management had a greater effect on the fungal communities of abundant and intermediate taxa than on the rare taxa. Available phosphorus (AP) and total nitrogen (TN) were the major determinants of fungal sub-communities in NT treatment. The abundant fungal sub-communities were assembled by deterministic processes under medium strength selection, while strong conservation tillage strength shifts the abundant sub-community assembly process from deterministic to stochastic. Overall, the investigation of the ecological network indicated that no-tillage and straw mulching practices decreased the complexity of the abundant and intermediate fungal networks, while not significantly influencing rare fungal networks. These findings refine our knowledge of the response of fungal sub-communities to conservation tillage management techniques and provide new insights into understanding fungal sub-community assembly.

## Introduction

Conservation tillage management, such as no-tillage (NT) or reduced tillage (RT) and straw retention, has been a widely-used practice in agriculture systems. Indeed, it is estimated that an area of 155 million hectares is subject to no-tillage management, accounting for 11% of all arable farmland worldwide (Kassam et al., 2014). Conservation tillage can conserve plant-available water and reduce soil erosion caused by rain, wind, and feed soil biota by increasing soil nutrient (Balesdent et al., 2000; Hobbs et al., 2008). On the other hand, soil microbes are crucial for nitrogen cycling and soil fertility enhancement, and are influenced by NT and straw mulching practices (Levy-Booth et al., 2014; Joergensen and Wichern, 2018).

Conservation tillage (e.g., straw mulching and NT) accumulates more C and N sources in the soil, and has the potential to minimize soil disturbance and enhance soil aggregation, thus creating a favorable soil nutrition condition for soil microbial communities (Guo et al., 2015; Wang et al., 2019). Long-term straw mulching and NT have been shown to significantly increase soil pH, total carbon and the C:N ratio (Wang et al., 2020a; Zhou et al., 2021); these environmental factors, in turn, can significantly influence microbial diversity. For example, soil pH is a strong predictor of microbial community diversity and structure (Hartmann et al., 2015). Whereas conservation tillage effects on soil properties have been well studied, however, there are still lack of effects of environmental factors on fungal community. In this study, we investigated the key regulatory factors of fungal sub-communities under different conservation tillage strategies.

In addition, the assembly process of the soil microbial community is crucial for understanding the response of ecosystems to environmental changes. In that regard, both stochastic processes and deterministic processes influence community assembly (Vellend, 2010; Tripathi et al., 2018). On the one hand, changes in the environmental conditions influence biotic and abiotic filtering and the structure of the microbial community deterministically (Chesson, 2000; Vellend, 2010). On the other hand, community assembly patterns arising from processes dispersal and ecological drift occur stochastically (Chave, 2004). The fungal communities in agricultural soils were strongly affected by stochastic processes (Jiao et al., 2021). In different tillage managements, assembly processes have been investigated in rhizosphere microorganism, including diazotrophic community (Li et al., 2021), arbuscular mycorrhizal fungi (Wang et al., 2020b), bacterial community (Wang et al., 2020c). However, stochastic and deterministic processes of fungal sub-communities under long-term conservation tillage management have not yet been clarified. Therefore, we sought to identify the assembly processes of fungal sub-communities across four different treatments.

Co-occurrence network analysis has been recently used to elucidate the potential complex interaction among different taxonomic group associated with patterns of assembly process (Li et al., 2021). Recent study has found that agricultural intensification decreased the complexity of fungal network, and the abundance of mycorrhizal fungi was highest under organic farming rather than no-tillage and conventional practices (Banerjee et al., 2019). Straw mulching has also been shown to reduce the complexity of fungal network, and increased the risk of root rot by increasing the abundance of the soil-borne pathogens *F. graminearum* and *F. moniliforme* (Wang et al., 2020a). Previous study reported that NT practices had higher densities of fungal mycelia than CT treatment (Beare et al., 1997). NT practices, meanwhile, may result in soil compaction, and plow tillage strengthens the fungal-fungal interactions and reduced tillage (RT) induces a more stable network structure than NT (Hu et al., 2021). The soil fungal sub-community co-occurrence patterns in long-term conservation tillage field remain unknown. In this paper, we compared the changes in the co-occurrence patterns of rare, intermediate and abundant sub-communities under different tillage and straw mulching practices.

Overall, while the effects of conservation tillage on fungal community diversity and functional group have been well documented (Degrune et al., 2016; Schmidt et al., 2019), there is still limited knowledge about fungal sub-communities. Furthermore, the research needs to pay more attention to the fact that microbial communities tend to consist of a few highly abundant taxa and numerous intermediate and rare taxa. It is therefore incomplete to analyze the microbial community in broad groups (e.g., at a domain or kingdom level); both abundant and rare groups should be considered if the community dynamics are to be fully understood (Jiang et al., 2019).

In this research, therefore, a field experiment applying fungal ITS region sequencing was conducted in a long-term conservation trial field at the Fengqiu National agro-ecological experiment station. We hypotheses that (i) conservation tillage managements create different environments for fungi, which increase the correlation between environment factors and fungal sub-communities, and deeply change fungi community structure and composition, (ii) the different ecological environments significantly alter the assembly processes of fungal sub-communities along with conservation tillage strength, (iii) different tillage and straw managements change the network structure for fungi sub-communities. The findings may provide a theoretical and practical foundation for sustainable agriculture development from a microbial ecology perspective.

## Materials and Methods

### Site Description and Soil Sampling

The study site is situated in Fengqiu National Agro-Ecological Experimental Station (35°00′N, 114°24′E), Chinese Academy of Sciences, Henan province, Central China. This area has a typical temperate continental monsoon climate, with an average annual temperature of 13.9°C and an average annual rainfall of 615 mm. The test soil is classified as Aquic Inceptisol, which is developed from the alluvial sediments of the Yellow River.

The long-term conservation field was commenced in 2006 and based on a completely randomized block design with three replications. The current experiment was set up in a maize-wheat crop rotation with four treatments: (1) tillage for wheat and no-tillage for corn (conventional tillage, CT), (2) tillage for wheat and no-tillage for corn plus straw mulching (CTS), (3) no-tillage for wheat and corn (NT), (4) no-tillage for wheat and corn coupling straw mulching (NTS). Regarding tillage practice, soils were plowed to 20–22 cm depth with a moldboard plow. Regarding straw mulching, residues were crushed into 2–3 cm pieces for maize and 6–7 cm pieces for wheat, and then were spread evenly on the soil surface as mulch. The amount of straw was related to the yield of the plot. As for no-straw mulching treatments, all residues were removed from the plots. Each experimental plot was 14 m × 6.5 m in size. Three soil samples were randomly collected from each plot from the surface layer of soil (0–20 cm) using a sterile soil driller. The three samples were immediately mixed to form a composite soil sample. The composite samples were sieved through a 2 mm sieve so as to homogenize them and remove plant roots and stones, before being transferred to the laboratory for storage at 4°C for measuring soil physicochemical properties, and at −80°C for DNA extraction.

### Analysis of Soil Physical and Chemical Properties

The physicochemical properties of all twelve soil samples were determined. Soil pH was determined using a pH meter (FE20-Five Easy PlusTM, Switzerland) with a 1:2.5 soil/water mixture. In addition, we measured the total organic carbon (TOC), total phosphorus (TP), alkaline nitrogen (AN), total nitrogen (TN), total potassium (TK), available phosphorus (AP), and available potassium (AK), following the method in Bao (2000).

### DNA Extraction and ITS Sequencing

Genomic DNA was extracted from approximately 0.5 g of fresh soil using a Fast DNA™ Spin Kit (MP Biomedicals, United States), following the manufacturer’s instructions. The quality of the extracted DNA was determined using a NanoDrop 2000 Spectrophotometer (Thermo Scientific, Wilmington, DE, United States). The fungal-specific ITS1 region was amplified with the ITS1F (CTTGGTCATTTAGGAAGTAA) and ITS2 (GCTGCGTTCTTCATCGATGC) primer sets (Mueller et al., 2014). The following PCR conditions were used: initial denaturation at 94°C for 30 s, 35 cycles consisting of 15 s of denaturation at 98°C, 30 s of annealing at 55°C, followed by 45 s at 72°C, and a final extension for 10 min at 72°C (Schmidt et al., 2019). Pooled PCR products were purified using the GeneJET™ Gel Extraction Kit (Thermo Scientific, United States). Finally, Personalbio Biotechnology Institute (Shanghai, China) sequenced the purified products using an Illumina Miseq platform (Illumina, United States).

### Processing of Sequence Analysis

The microbiome sequences was processed using QIIME pipeline (Version 1.9.0) (Caporaso et al., 2012), and low-quality sequencing reads with a length shorter than 150 bp, and with an average base quality score < 20 were discarded from further analysis. Operational taxonomic units (OTUs) were clustered at a ≥ 97% similarity level using the UCLUST feature in QIIME 1 (Edgar et al., 2011). Taxonomic identification of representative sequences was performed using the BLAST database. Genomic sequencing data has been deposited in the NCBI Sequence Read Archive (BioProject ID PRJNA764374, submission ID SUB10399701).

### Statistical and Bioinformatics Analysis

We defined rare, intermediate and abundant fungal sub-communities to support the understanding of fungal community variation. OTUs with relative abundance above 0.5% were defined as “abundant,” while those below 0.01% were defined as “rare.” Those taxa with relative abundance between 0.5 and 0.01% OTUs were defined as “intermediate.” We calculated the relative abundance of these sub-communities across all samples. This definition was based on previous research (Liu et al., 2015; Jiang et al., 2019).

Alpha-diversity indices (Shannon and Chao1) were calculated using MOTHUR. Significant differences in α- diversity and in soil properties were analyzed by ANOVA using SPSS (Version 21.0, SPSS, Chicago, IL, United States). Fungal sub-community alpha-diversity indices were calculated using the R package “vegan,” and analyzed by one-way ANOVA in SPSS.

Volcano plots were used to show the differential abundance of OTUs. We selected the false discovery rate (FDR) to adjust *p*-values (Love et al., 2014). The log_2_ Fold Change (log_2_FC) and adjusted *p*-values were calculated using the R package “DESeq2.” The OTUs’ differential abundance plots were constructed using the R package “ggplot2.” PCA was performed using the “Principal Component analysis” application in the OriginPro 2021 (Version 9.80).

Linear discriminant analysis (LDA), combined with effect size (LEfSe) measurements, were performed in order to find statistical biomarkers between treatments (Segata et al., 2011). This analysis was conducted on the Hutlab Galaxy website application^1^. Spearman correlations in R (Version 4.1.1) were used to evaluate the relationship between various environmental factors. The correlation between fungal sub-communities and environmental factors were normalized by using the Mantel test (Diniz-Filho et al., 2013).

The assembly processes of fungal sub-communities were constructed using the “ses.comdistnt” function in the “MicEco” package (Stegen et al., 2012), with the beta mean nearest taxon distance (βMNTD) metric used to determine turnover in the phylogenetic structure of community. Meanwhile, the stochastic and deterministic ecological processes of fungal sub-communities were evaluated through null model analysis (Stegen et al., 2012). This method uses randomizations to estimate the standard deviation of the observed βMNTD compared to a null distribution (999 randomizations) for each βMNTD estimate. The β-nearest taxon index (βNTI) was used to evaluate the deviation between the mean of the null βMNTD and observed βMNTD, expressed in units of standard deviations (Stegen et al., 2013).

In order to analyze the fungal assembly processes quantitatively, we determined the proportion of dispersal limitation and undominated for stochastic processes, and variable selection and homogeneous selection for deterministic processes. This was done in the “iCAMP” R package (Ning et al., 2020). Variations were investigated by comparing β-diversity metrics (βNTI values and RC_bray_). Meanwhile, the influences of the variable selection and homogeneous selection fractions were determined according to thresholds of βNTI values > 2 and < −2, respectively. The relative influence of dispersal limitation was quantified as a pairwise comparison between |βNTI| < 2 and RC_bray_ > 0.95, whereas |βNTI| < 2 and RC_bray_ < 0.95 was used to estimate the influence of undominated process (Stegen et al., 2013, 2015).

Microbial networks were created to construct the interaction networks for tillage (CT and CTS) treatments and no-tillage (NT and NTS) treatments, straw mulching (CTS and NTS) and no-straw mulching (CT and NT). In our study, abundant, intermediate and rare OTUs were used for network construction. The Spearman’s rank correlation coefficients were calculated using the R package “psych.” The correlation coefficients (*P* < 0.05 and *r* > 0.6) were corrected for analysis of network. The network graphs were visualized and the topological properties of the network were calculated using Gephi software (Version 0.9.2^2^). OTUs in the networks were identified as potential plant pathogens using FUNGuild tool (Nguyen et al., 2016).

## Results

### Richness and Diversity Indices of Fungal Communities

**FIGURE 1:**
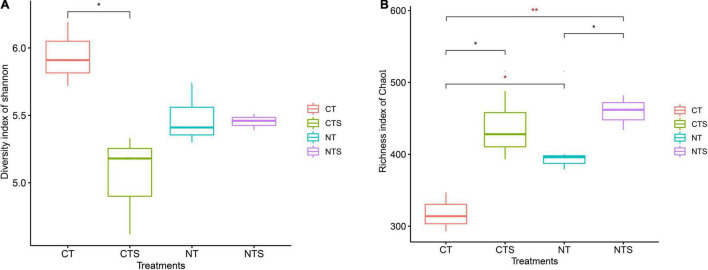
Fungal alpha diversity associated with different soil treatments. The indices of diversity and richness are shown as Shannon **(A)** and Chao1 **(B)**, respectively. Statistically significant differences between different treatments were determined by *T*-test ANOVA (*p* < 0.05). The symbols *, ** are used to show statistical significance at the 0.05, 0.01 level, respectively.

The Shannon estimator for fungal diversity was found to be significantly greater in conventional tillage (CT) than in no-tillage and straw mulching treatments (CTS, NT, and NTS). While the Chao1 richness index was markedly lower in the conventional treatment (CT) than in the conservation tillage groups (CTS, NT, and NTS) (Figure 1). We discovered that the richness indices had significantly higher values in the plots subject to straw retention practices (CTS and NTS) than in those subject to other tillage practices (CT and NT).

### Depleted and Enriched Operational Taxonomic Units Response to Different Tillage Systems, and Variations of Fungal Community Structure

**FIGURE 2:**
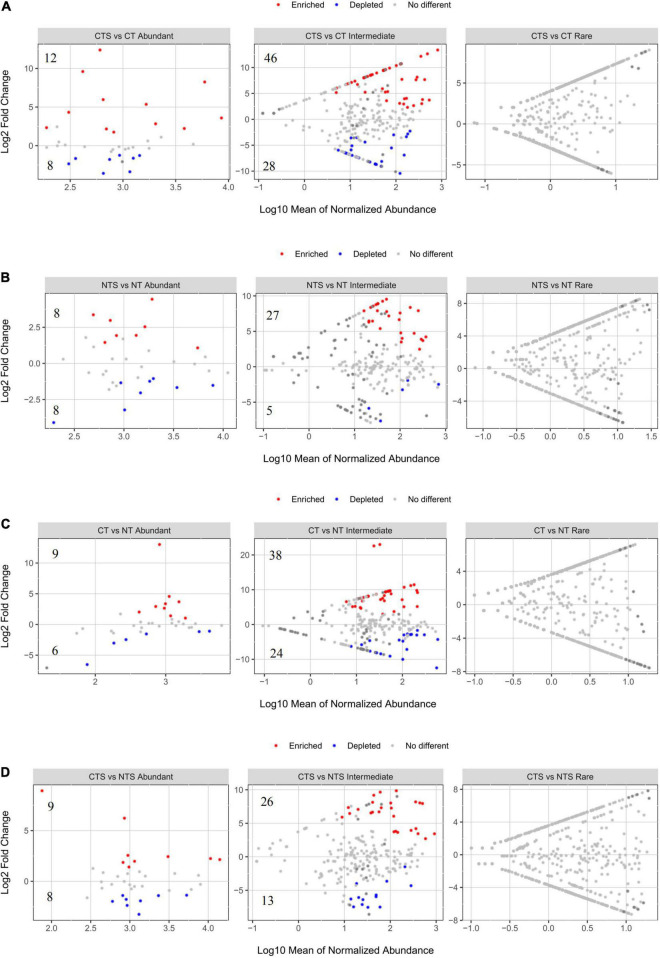
Volcano plots illustrating OTUs significantly enriched (red) and depleted (blue) by straw mulching **(A,B)** and tillage **(C,D)** managements for fungal sub-communities. Each point indicates an individual OTU.

We conducted groups comparison analysis to identify OTUs where abundance was strongly influenced by straw mulching (CTS vs. CT and NTS vs. NT) and tillage (CT vs. NT and CTS vs. NTS). OTUs where relative abundance significantly increased or decreased were referred to as “Enriched OTUs” and “Depleted OTUs,” respectively. These enriched and depleted OTUs were found to occur only in abundant and intermediate taxa (Figure 2). There were, respectively, 12 and 46 enriched OTUs in the “CTS vs. CT” group, and 8 and 27 enriched OTUs were in the “NTS vs. NT” group (Figures 2A,B). Thus, there were more enriched than depleted OTUs under straw mulching practice. Furthermore, both abundant and intermediate fungal taxa exhibited more enriched than depleted OTUs in the tillage comparison groups (Figures 2C,D).

Principle component analysis (PCA) of the fungal sub-communities among the four treatments showed that the structure of abundant and intermediate taxa communities were obviously distinct (Supplementary Figure 1). Our results indicated that conservation tillage significantly affected the abundant and intermediate taxa communities. In respect to the abundant taxa, the two principal components account for 54.4% of the total variance. In contrast, in rare taxa, the two principle components account just 33.2% of the total variance.

### Fungal Communities With Statistically Significant Differences

**FIGURE 3:**
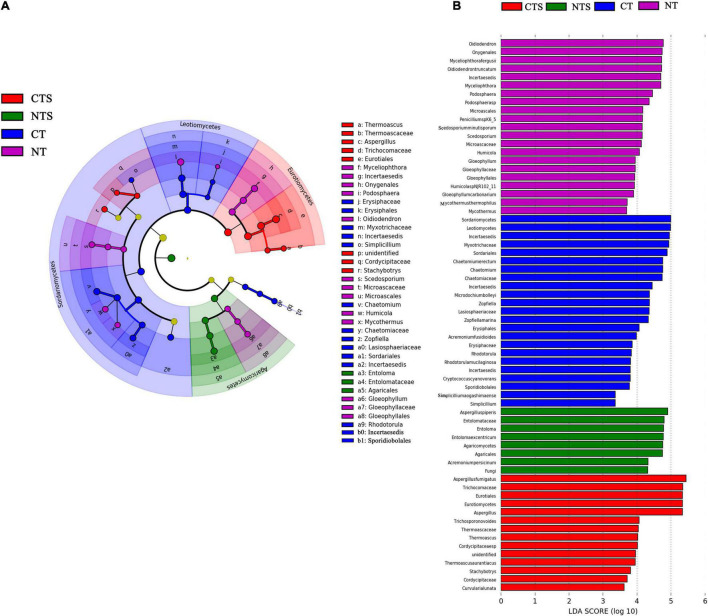
Cladogram showing the phylogenetic distribution of the fungal lineages associated with soil from the four treatments **(A)**. Indicator fungi with LDA scores > 3 **(B)**. Different colors represent different treatments (red, CTS; green, NTS; blue, CT; purple, NT). The circle from inside to outside represents phylogenetic levels from domain to genus, with its diameter reflecting the relative abundance of fungi.

The Cladogram in Figure 3 shows the phylogenetic distribution of fungal lineages that were markedly associated (LDA value > 3) with samples from different tillage management fields (Figures 3A,B). LEfSe was applied to find statistically different biomarkers among four treatments. In the CTS treatment, three groups were significantly enriched, namely *Eurotiales* (from order to genus), *Cordycipitaceae* (from family to genus) and *Stachybotrys* (genus). In the NTS treatment, the enriched fungi was *Agaricomycetes* (from class to genus). In the CT treatment, the fungal taxa were mostly enriched at the family level, including *Myxotrichaceae*, *Erysiphaceae*, *Chaetomiaceae*, *Incertaesedis*, and *Lasiosphaeriaceae*. In the NT treatment, six groups were found to be significantly enriched, namely *Onygenales* (from order to genus), *Oidiodendron* and *Podospora* (genus), *Microascales* (from order to genus), *Gloeophyllales* (from order to genus), *Chaetomiaceae* (its genus *Humicola* and *Mycothermus*) (Figure 3A).

### Correlation of Fungal Sub-Communities With Environmental Factors

As illustrated in Supplementary Table 1, TOC, TN, AN, AP, and AK were increased in straw mulching treatments (CTS and NTS) compared to no-straw treatments (NT and CT), indicating that straw mulching probably helps to accumulate nutrition in the soil layer. No-tillage (NT and NTS), meanwhile, increased the content of TP compared to tillage (CT and CTS) treatments.

**FIGURE 4:**
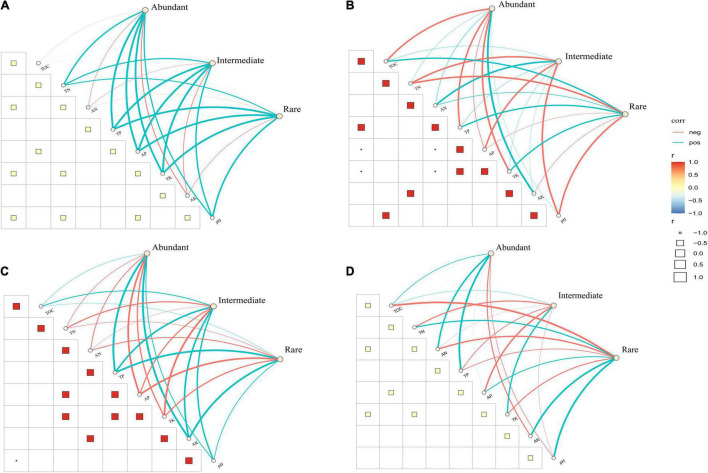
Pairwise comparisons of soil properties and their effects on fungal sub-communities composition in four treatments, with a color gradient denoting Spearman’s correlation coefficients. Edges represent Mantel’s r for correlations, and the color corresponding to the significance. **(A)** CT. **(B)** CTS. **(C)** NT. **(D)** NTS.

In the CT treatment, most of the soil properties were negatively correlated with fungal sub-communities (Figure 4A). The results showed that AP has a positive correlation with abundant, intermediate and rare taxa, TN and pH significantly influence the composition of intermediate and rare taxa in the CTS treatment (Figure 4B). In the NT treatment, three fungal sub-communities were simultaneously influenced by multiple factors including TN, AN, AP and TK (Figure 4C). As for NTS treatment, environmental factors (TOC, TN, AN, TP, AP and TK) mainly had a positive influence on intermediate and rare taxa (Figure 4D). Overall, AP and TN were the strongest correlates of fungal sub-communities in the conservation tillage treatments.

### Assembly Processes in Abundant, Intermediate and Rare Fungal Sub-Communities

**FIGURE 5:**
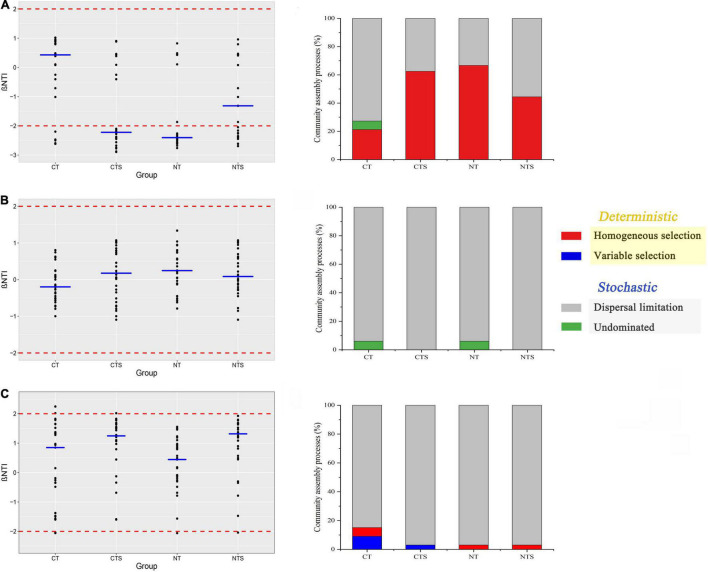
Scatter plot of βNTI values and quantitative analysis of the assembly processes that govern the turnover of abundant **(A)**, intermediate **(B)** and rare **(C)** taxa communities in four treatments. The blue line represents the median values of each treatment.

Briefly, |βNTI| > 2 and |βNTI| < 2 represent the deterministic and stochastic processes, respectively. With respect to abundant fungal taxa, deterministic processes comprised more than 62.5% of the assembly processes in CTS and NT treatments, while stochastic processes comprised more than 55.5% of the processes shaping abundant sub-communities in CT and NTS treatments (Figure 5A). The distribution of βNTI values across all treatments showed that stochastic processes comprised more than 84.8% of the assembly processes shaping intermediate and rare taxa (Figures 5B,C).

We also used quantitative analyses to explain the assembly processes of fungal sub-communities more fully. Dispersal limitation contributed the largest fraction to the assembly of both rare (>84.8%) (Figure 5C) and intermediate sub-communities (>93.9%) (Figure 5B). Homogeneous selection (> 62%) contributed a large fraction to the assembly of abundant sub-communities in CTS and NT treatments, followed by dispersal limitation. By contrast, in NTS and CT treatments, dispersal limitation (> 55%) contributed a lager fraction to the assembly of abundant sub-communities than homogeneous selection (Figure 5A). The undominated process (6.06%), variable selection (< 9%) contributed smaller fractions to the fungal assembly processes (Figures 5B,C).

### Tillage and Straw Mulching Practices Changed the Fungal Co-occurrence Patterns

**FIGURE 6:**
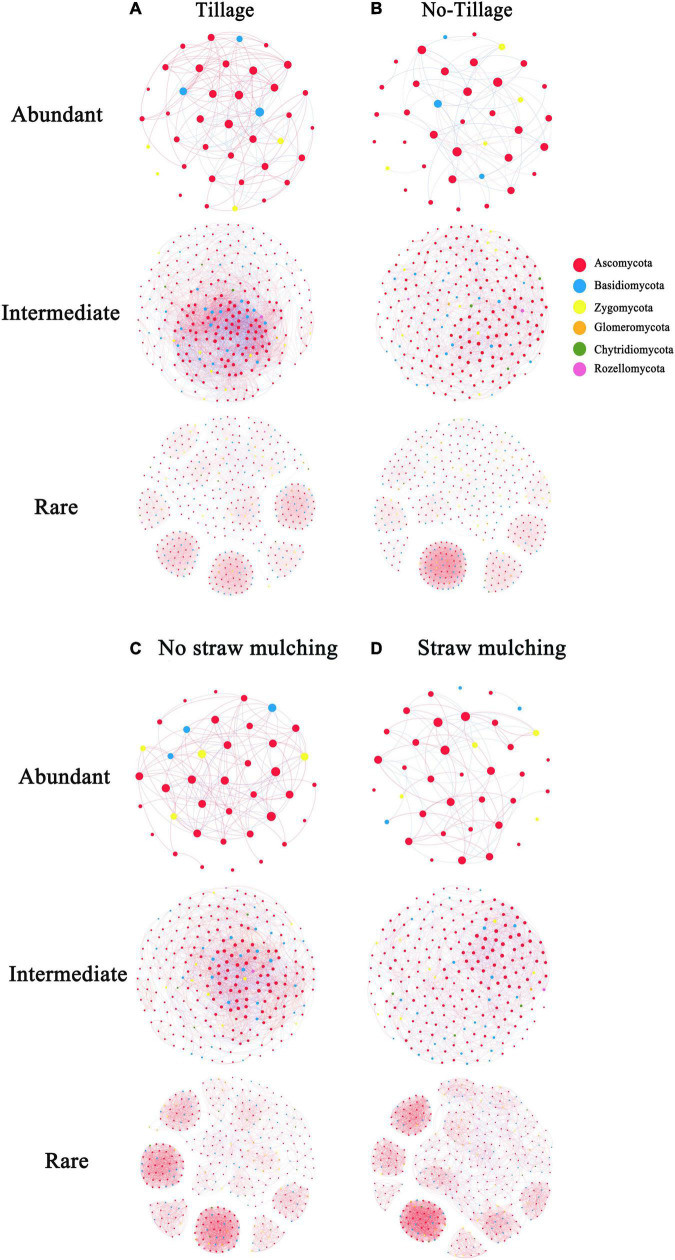
Network co-occurrence analysis (Spearman’s ρ > 0.6 and significant *p* < 0.05) of fungal sub-communities in the tillage (CT and CTS) and no-tillage (NT and NTS) treatments, no-straw mulching (CT and NT) and straw mulching (CTS and NTS). The size of each node is proportional to the relative abundance; red lines and blue lines represent positive and negative correlations, respectively; nodes of the same color belongs to the same phylum. **(A)** Tillage (CT and CTS). **(B)** No-tillage (NT and NTS). **(C)** No straw mulching (CT and NT). **(D)** Straw mulching (CTS and NTS).

We used co-occurrence network analysis to reveal the complexity of fungal sub-community networks (Figure 6). The fungal empirical co-occurrence patterns differed significantly with the application of the different tillage practices. As revealed by the network parameters (Supplementary Table 2), both abundant and intermediate fungal taxa had less complexity in no-tillage treatments than in tillage treatments. The tillage treatments increased the number of nodes and edges compared to no-tillage treatments for abundant and intermediate taxa. The number of edges increased by 2.5-fold (the sum of abundant and intermediate taxa) in tillage treatments, indicating the strong interaction in the abundant and intermediate fungal sub-communities. In respect to rare taxa, however, there were no significant changes in the number of nodes and edges in tillage treatments compared to no-tillage treatments. Straw mulching, meanwhile, reduced the complexity of the abundant and intermediate taxa network, as reflected by the lower number of nodes and edges (Supplementary Table 2).

Tillage management was also shown to have different effects on the topological properties of fungal sub-communities. The average clustering coefficient and average degree decreased, but the average path length increased in the no-tillage treatment for abundant and intermediate taxa, while the changes in these topological properties were not significant for the rare taxa. These results showed that the tillage practice notably increased the proportions of positive links in the abundant taxa (61.1% in tillage and 45.3% in no-tillage). The proportions of positive links in abundant taxa were also increased in no-straw mulching (57.7%) compared to straw mulching (43.8%). Our result showed that no-tillage practices had more abundances of potential plant pathogens than tillage practices. Straw mulching had the highest abundance of potential plant pathogens among four practices, and these pathogens mainly have positive correlation with other fungi in the networks (Supplementary Table 3).

## Discussion

### Effects of Conservation Tillage on Fungal Alpha Diversity

Our finding indicated that conservation tillage practices significantly increased fungal richness, but no significant effect on fungal diversity, which is consistent with previous research (Wang et al., 2020a). In this study, we found that conservation tillage (CTS, NTS, and NT) has higher organic carbon content than CT (Supplementary Table 1), previous study indicated that fungal richness was significantly correlated to soil organic carbon (SOC) content, thereby contributing to soil fungal richness (Yang et al., 2012). Additionally, a previous study demonstrated that conservation tillage practices have no favorable effect on fungal community diversity (Degrune et al., 2016). A study noted that conservation tillage management can lead to greater fungal diversity by changing soil microenvironment (Wang et al., 2017). This disparate understanding of the effect of conservation tillage on the soil fungal community may be due to the complexity of the environmental conditions. For example, soil with high clay and sand fractions can lead to modification in fungal community (Bach et al., 2010). Soil management histories (forest to cultivated land vs. longstanding cultivation) are also key factors (Degrune et al., 2016).

### Straw Mulching and Conventional Tillage Affected the Fungal Community Structure

We picked out enriched and depleted OTUs to analyze the differences in the fungal sub-communities using differential abundance analysis. We observed that abundant and intermediate taxa had enriched and depleted OTUs except rare taxa. Compared to CT treatment, CTS increased the proportion of enriched OTUs more than depleted OTUs in abundant and intermediate taxa. NTS, meanwhile, increased the proportion of enriched OTUs more than depleted OTUs. These results indicate that straw mulching helps fungi to grow. Organic farming practice has positive effect on fungi biomass, probably because carbon content is the key factor that governing microbial growth (Birkhofer et al., 2008; Wang et al., 2017). Although NT can provide more favorable conditions (a cooler and moist environment) than CT (Helgason et al., 2009), our results showed that CT enriched more OTUs than NT. Soil disturbance can improve the distribution of plant residues and substrate availability, distributing soil aggregates and releasing particulate organic matter, and thus supporting the growth of micro-biota (Chaer et al., 2009).

Conservation tillage can affect residue decomposition and alter gas and water movement, leading to changes in fungal community patterns (Holland, 2004; Wang et al., 2016, 2017). The PCA results showed that conservation practices significantly influence on abundant and intermediate taxa, but do not have a significant influence on rare taxa community structure. In our study, the total variance explained by PCA was much higher for the abundant sub-community (54.4%) than for the rare sub-community (33.2%). Rare microbes have a large proportion of unexplained variation because rare taxa are more subject to biotic interaction (e.g., competition) and have discrepant ecological niches (Liu et al., 2015; Lopes and Fernandes, 2020).

We used LEfSe analysis to understand the variation in fungal communities in long-term conservation fields more fully. This method can analyze the microbial community at any clade. We retained the taxa with significant differences and filtered out those without significant differences. Statistical analysis was performed from phylum to genus.

According to the LEfSe results, *Eurotiales* were enriched in CTS treatment. A recent study has demonstrated that organic matter can promote the growth of fungal taxa, and *Eurotiales* is important for the SOC decomposition process (Wang et al., 2021). One of the other fungi found to be enriched in the plots subject to NTS treatment was *Agaricomycetes*, which can degrade various substrates, such as cellulose and lignin (Zhang et al., 2021). Decomposition of crop residues on the soil surface could therefore be enhanced by this fungal growth. In addition, a recent study has shown that *Scedosporium*, which is considered to have pathogenic potentials, is enriched in NT treatment (Kitisin et al., 2021). On the other hand, Wang et al. (2020a) found that NT may increase the risk of stubble-borne diseases.

### Environmental Drivers of Fungal Sub-Communities Under Conservation Practice

No-tillage and straw mulching have been found to have a significant effect on soil nutrient parameters (Wang et al., 2020a). Our results showed that NT significantly increased the soil TP content, probably on the basis that it increases the residual P concentration in the soil surface layer (Jansa et al., 2003). Furthermore, crop residual acts as a carbon source as well as increasing the organic matter content of soil (Bu et al., 2020). We also observed that TOC, TN, and AN contents increased under straw mulching treatments, which is in line with a previous study reporting that the use of cover crops accounted for most of the N increase associated with crop rotation effects (McDaniel et al., 2014). Overall, conservation tillage treatment improves nutrition conditions for soil microbial communities.

Previous studies have revealed that pH and nutrient levels are key predictors of fungal composition (Lauber et al., 2008; Rousk et al., 2010). Our findings support this by showing that AP, TN and AN simultaneously influenced the fungal sub-communities in NT treatment. In the conservation tillage treatments (CTS, NT and NTS), soil properties were closely related to fungal sub-communities. In contrast, there was a mainly negative relationship between fungal sub-communities and soil properties in CT treatment.

### Different Assembly Processes Experienced by Abundant, Intermediate and Rare Fungal Sub-Communities

Uncovering the underlying microbial assembly processes is a key subject for microbial ecology (Nemergut et al., 2013). It is generally recognized that spatial heterogeneity and environmental filtering contribute to the microbial assembly and community structure (Xue et al., 2021). Our results showed that, in CTS and NT treatments, the assembly of abundant fungal taxa was governed by deterministic processes, whereas in CT treatment sub-community assembly was governed by stochastic processes. A significant correlation between environment factors and fungal sub-communities was found in NT and CTS. Specifically, it was revealed that no-tillage and straw mulching positively influenced the soil properties and this shaped the abundant sub-community.

The assembly of abundant taxa in plots subject to NTS treatment, however, was governed by stochastic processes, and the abundant taxa diversity indices (Shannon) were higher in NTS plots than those subject to CTS or NT treatments (Supplementary Table 4). NTS can therefore serve to increase the diversity of the fungal community, probably by increasing the total C and bioavailable C (Navarro-Noya et al., 2013). High-diversity communities are dominated by stochastic processes, while low diversity ones depended on deterministic processes which constrained the community function (Xun et al., 2019), which in line with our findings. Similarly, intermediate and rare fungal taxa were dominated by stochastic processes, while abundant taxa exhibited a remarkably wide response to the ecological preferences in agriculture fields (Jiao and Lu, 2020a).

Previous studies have indicated that the assembly processes of different sub-communities rely on different environmental factors in the agro-ecosystem (Jiao et al., 2017; Jiao and Lu, 2020b). Homogeneous selection significantly affected the abundant sub-communities in NT and CTS treatments, whereas rare and intermediate sub-communities were more subject to dispersal limitations. This contrasts with previous research appearing to show that rare sub-communities are governed mainly by homogeneous selection (Jiao and Lu, 2020a). These discrepancies may be caused by straw retention and no-tillage practices and by geography (Shi et al., 2018). The dominant status of homogeneous selection in CTS and NT plots suggests that abundant taxa are more sensitive to conservation practice, while the fact that dispersal limitation dominated the rare and intermediate taxa implied a weak link with no-tillage and straw retention practices.

**FIGURE 7:**
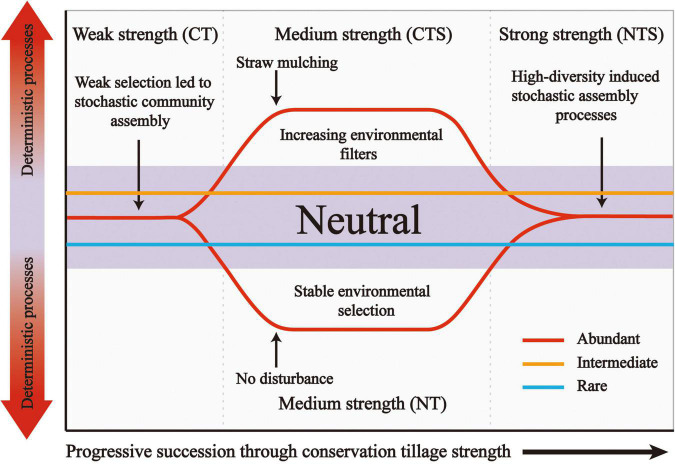
Conceptual model showing how ecological selection dominates the structure of fungal sub-communities through conservation tillage strength. Weak conservation tillage strength, CT; Medium conservation tillage strength, CTS and NT; Strong conservation tillage strength, NTS.

To understand the assembly processes of fungal sub-communities more fully, we established a conceptual model (Figure 7). This presents that ecological processes can emerge in the following forms: (i) under weak strength selection (conventional tillage conditions), the establishment of fungal sub-communities is dominated by stochastic processes, (ii) under medium strength selection (straw mulching or no-tillage practices), changes in environment conditions enhance the selection leading to deterministic processes dominating in the abundant sub-community, (iii) strong strength selection (straw mulching combined with no-tillage) increase the diversity of the abundant fungal sub-community and thus induced stochastic processes. Notably, intermediate and rare sub-communities are consistently dominated by stochasticity. These microbial taxa are characterized by high levels of organismal dispersal, and influenced by stochastic birth or death rather than environmental filters (Dini-Andreote et al., 2015).

### No-Tillage and Straw Mulching Practices Changed the Co-occurrence Patterns of Abundant and Intermediate Taxa

In the present study, we observed that no-tillage and straw mulching reduced the complexity of the abundant and intermediate fungal taxa network, while conservation tillage practices had no significant influence on the rare taxa network. Previous studies showed that agricultural intensification reduced the complexity of the microbial network, and tillage practice was considered to be harmful to the extension of fungal mycelia (Caesar-TonThat et al., 2010; Banerjee et al., 2019). Our results, however, showed that conventional tillage increased the fungal interaction and the proportion of positive link in abundant taxa compared to conservation tillage treatments.

Our results might be explained by recognizing that microbial ecology is affected by nutrient availability, aeration, moisture and pH (Fierer and Jackson, 2006). It may be that the full soil inversion created by tillage practice can promote soil nutrients used by fungi and strengthen the links in the microbial network (Six et al., 2000; Le Guillou et al., 2012). Furthermore, another recent study has found that the abundance of arbuscular mycorrhizal fungi (AMF) was higher in conventional than in conservation tillage, which the authors explained by the dilution of P in the surface soil layer in tillage practice (Lopes and Fernandes, 2020). Wang et al. (2020a) found that straw mulching decreased both fungal and bacterial network complexity, possibly due to straw mulching created favorable nutrient conditions for fungi, decreasing microbial inhibition and competition, and thus weakening interaction and negative relevance (Bronstein, 1994; Cao et al., 2018). These results indicated that conventional tillage practice can deliver a more stable fungal network in corn-wheat rotation systems. In our study, compared to tillage practices, NT practices had more abundances of potential plant pathogens. Straw mulching practices had more abundances of plant pathogens than no-straw mulching practices. Conservation tillage can enhance the growth of plant pathogens by concentrating plant debris, and tillage practices might alleviate the ecological risks of the pathogens (Sturz et al., 1997; Hu et al., 2021). We observed that potential plant pathogens mainly have positive correlation with other fungi in the networks, might due to the cooperation of fungi in the decomposition of straw residues (Hu et al., 2021).

## Data Availability Statement

The datasets presented in this study can be found in online repositories. The names of the repository/repositories and accession number(s) can be found below: Genomic sequencing data has been deposited in the NCBI Sequence Read Archive (BioProject ID PRJNA764374, submission ID SUB10399701).

## Author Contributions

HC and AZ revised the manuscript and developed the experiment idea. CZ and HL conducted most of the experiments, prepared the manuscript, and analyzed the data. SL analyzed the data and revised the manuscript. SH contributed to supervision. LZ, XY, KC, and XX participated in revision of the manuscript. All authors read and agreed the final manuscript.

## Conflict of Interest

The authors declare that the research was conducted in the absence of any commercial or financial relationships that could be construed as a potential conflict of interest.

## Publisher’s Note

All claims expressed in this article are solely those of the authors and do not necessarily represent those of their affiliated organizations, or those of the publisher, the editors and the reviewers. Any product that may be evaluated in this article, or claim that may be made by its manufacturer, is not guaranteed or endorsed by the publisher.

## Footnotes

1
http://huttenhower.sph.harvard.edu/galaxy/

2
https://gephi.org/
