# Supplementary material for: Response of Fungal Sub-Communities in a Maize-Wheat Rotation Field Subjected to Long-Term Conservation Tillage Management
Source: Front Microbiol. 2022 Mar 29;13:829152. doi: 10.3389/fmicb.2022.829152 (PMC9002332; doi:10.3389/fmicb.2022.829152)
Supplement: Supplementary file 1 [file Data_Sheet_1.docx]

**Supplementary table 1.** Physico-chemical properties of the soils in different treatments

| Treatment | TOC (g kg^-1^) | | TN (g kg^-1^) | AN (mg kg^-1^) | TP (g kg^-1^) | AP (mg kg^-1^) | TK ( g kg^-1^) | AK (mg kg^-1^) | pH |
| --- | --- | --- | --- | --- | --- | --- | --- | --- | --- |
| CTS | 13.88±0.06^a^ | 0.95±0.06^a^ | | 76.14±1.55^a^ | 0.68±0.01^d^ | 17.65±1.14^a^ | 21.54±0.34^a^ | 147.05±8.14^a^ | 7.89±0.18^a^ |
| NTS | 14.61±0.42^a^ | 0.97±0.01^a^ | | 75.59±1.34^a^ | 0.89±0.01^b^ | 14.99±0.40^a^ | 21.99±0.71^a^ | 160.35±16.95^a^ | 8.11±0.03^a^ |
| CT | 12.81±0.63^a^ | 0.85±0.08^ab^ | | 60.80±2.32^b^ | 0.79±0.01^c^ | 15.36±1.84^a^ | 21.65±0.75^a^ | 77.22±0.00^b^ | 8.01±0.12^a^ |
| NT | 13.11±0.87^a^ | 0.81±0.01^b^ | | 65.18±2.05^c^ | 0.93±0.01^a^ | 13.93±1.29^a^ | 21.75±0.42^a^ | 77.22±0.00^b^ | 8.07±0.04^a^ |

**Note:** Total organic carbon, TOC; Total nitrogen, TN; Alkaline nitrogen, AN; Total phosphorus, TP; Available phosphorus, AP; Total potassium, TK; Available potassium, AK; Values in the same column followed by different letters differ significantly (P < 0.05).

**Supplementary table 2.** Topological properties of the fungal sub-community networks

|  | Tillage (CT and CTS) | | | No-tillage (NT and NTS) | | |
| --- | --- | --- | --- | --- | --- | --- |
|  | Abundant | Intermediate | Rare | Abundant | Intermediate | Rare |
| Number of nodes^a^ | 39 | 250 | 372 | 38 | 222 | 376 |
| Number of edges^b^ | 144 | 4523 | 5334 | 75 | 1751 | 5136 |
| Positive edges^c^ | 88 | 2431 | 4866 | 34 | 944 | 4879 |
| Negative edges^d^ | 56 | 2092 | 468 | 41 | 807 | 437 |
| Modularity^e^ | 1.135 | 3.49 | 0.867 | -12.2 | 4.14 | 0.684 |
| Number of community^f^ | 11 | 12 | 14 | 13 | 10 | 14 |
| Network diameter^g^ | 7 | 6 | 9 | 6 | 5 | 6 |
| Average path length^h^ | 2.525 | 2.497 | 2.8 | 2.685 | 2.859 | 2.795 |
| Average degree^i^ | 7.385 | 36.184 | 28.677 | 3.974 | 15.775 | 28.227 |
| Average clustering coefficient^j^ | 0.659 | 0.617 | 0.861 | 0.503 | 0.518 | 0.82 |
| Density^k^ | 0.194 | 0.145 | 0.077 | 0.107 | 0.071 | 0.075 |
|  | No straw mulching (CT and NT) | | | Straw mulching (CTS and NTS) | | |
|  | Abundant | Intermediate | Rare | Abundant | Intermediate | Rare |
| Number of nodes^a^ | 39 | 241 | 340 | 38 | 224 | 372 |
| Number of edges^b^ | 130 | 2794 | 4101 | 80 | 1814 | 4203 |
| Positive edges^c^ | 76 | 1529 | 3810 | 35 | 950 | 3783 |
| Negative edges^d^ | 54 | 1265 | 291 | 45 | 864 | 420 |
| Modularity^e^ | 1.91 | 3.149 | 0.843 | -10.063 | 7.748 | 0.868 |
| Number of community^f^ | 11 | 9 | 14 | 16 | 9 | 14 |
| Network diameter^g^ | 6 | 7 | 8 | 5 | 6 | 6 |
| Average path length^h^ | 2.349 | 2.726 | 2.847 | 2.243 | 2.912 | 3.026 |
| Average degree^i^ | 6.667 | 23.187 | 24.124 | 4.211 | 16.196 | 22.597 |
| Average clustering coefficient^j^ | 0.582 | 0.592 | 0.855 | 0.749 | 0.541 | 0.795 |
| Density^k^ | 0.175 | 0.097 | 0.071 | 0.114 | 0.073 | 0.061 |

**Note**: CT, soil sampled from convention tillage field. CTS, soil sampled from tillage and straw mulching field. NT, soil sampled from the site of no-tillage field. NTS, soil sampled from the site of no-tillage and straw mulching field. Fungal OTUs with significant (p < 0.05) and strong (r >0.6) correlation. Correlation analysis was performed by R language (R 2021, version 4.1.1). The parameters of network were calculated by Gephi 0.9.2.

^a,b^Number of connection obtained by Gephi.

^c^Positive correlation between two fungal OTUs.

^d^Negative correlation between two fungal OTUs.

^e^Structure with high-density connection between nodes.

^f^A community is defined as a group of nodes that are densely connected internally.

^g^The longest distance between nodes in the network.

^h^Average network distance between all pairs of nodes or the average length of all edges.

^i^The average number of connections of every node in the network .

^j^The average clustering coefficient is defined as the mean value of individual coefficients.

^k^The density used to measure how close the network is to complete. A complete graph has all possible edges and density equal to 1.

**Supplementary table 4.** α-diversity of fungal sub-communities

| Fungal  Sub-communities | Treatment | Shannon | Chao1 |
| --- | --- | --- | --- |
| Abundant | CTS | 3.94±0.33^a^ | 36.67±0.58^a^ |
|  | NTS | 4.38±0.10^a^± | 36.67±0.58^a^ |
|  | CT | 4.21±0.62^a^± | 37.00±1.00^a^ |
|  | NT | 4.17±0.09^a^ | 36.33±0.58^a^ |
| Intermediate | CTS | 4.06±0.10^a^ | 196.92±13.25^a^ |
|  | NTS | 4.21±0.16^a^ | 213.89±34.20^a^ |
|  | CT | 3.89±0.39^a^ | 137.00±7.55^b^ |
|  | NT | 3.95±0.05^a^ | 173.57±4.74^a^ |
| Rare | CTS | 6.05±0.43^a^ | 218.55±84.27^a^ |
|  | NTS | 6.32±0.66^a^ | 217.30±16.29^a^ |
|  | CT | 6.20±0.53^a^ | 145.86±19.23^a^ |
|  | NT | 5.93±0.20^a^ | 182.88±12.44^a^ |

**Note**: Values in the same column followed by different letters differ significantly (P < 0.05)


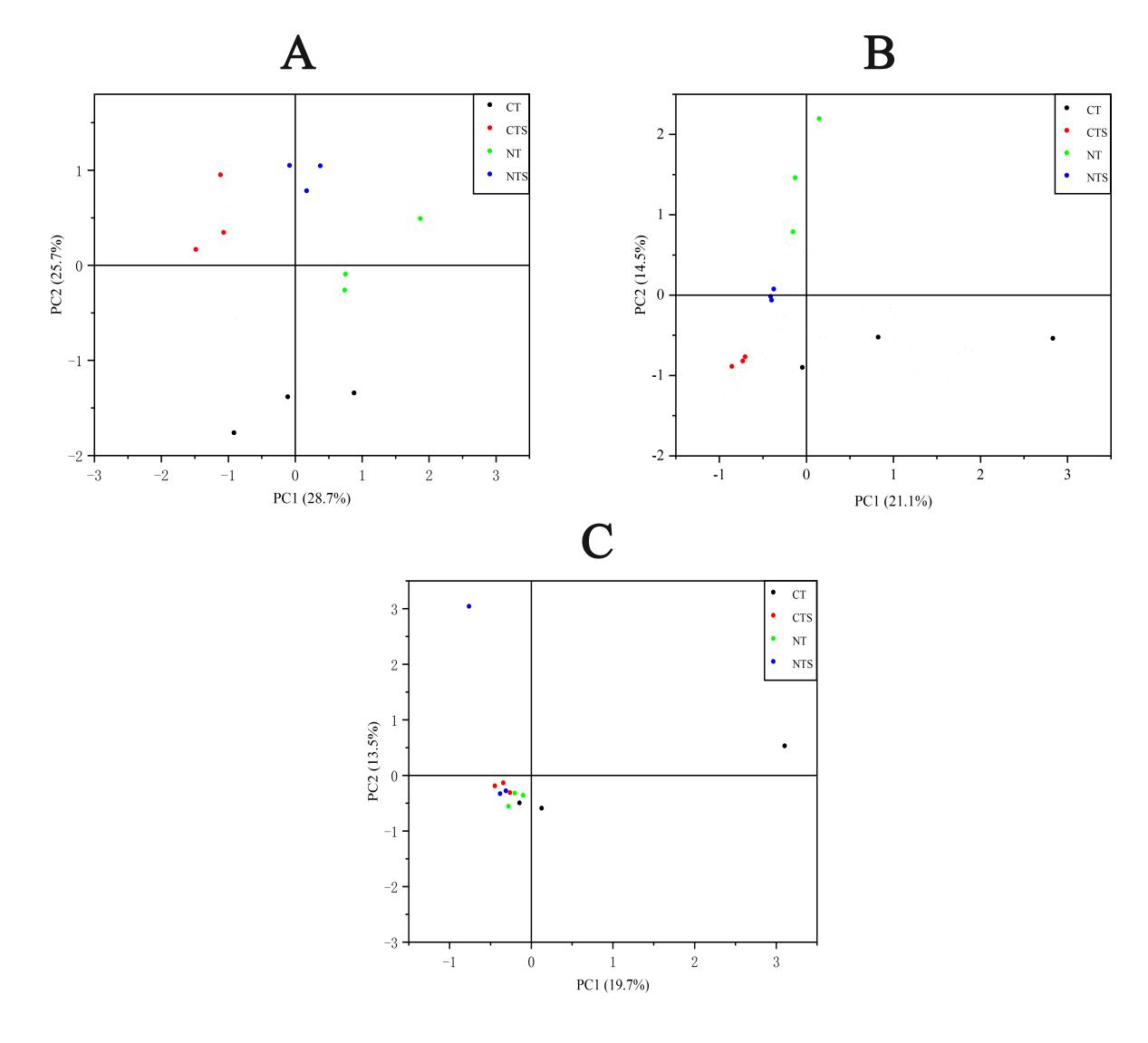


**Figure. S1**. Principal component analysis of the abundant (A), intermediate (B) and rare (C) sub-communities in four treatments.
